# Strengthening the sustainability of neglected tropical disease programs in Rwanda: An assessment of access and utilization of domestically-financed services for soil-transmitted helminthiases and schistosomiasis

**DOI:** 10.1371/journal.pntd.0012371

**Published:** 2025-08-29

**Authors:** Urbanus Kioko, Eugene Ruberanziza, Sam Macintosh, Donatien Ngabo, Vincent Okungu

**Affiliations:** 1 Axis Health Analytics, Nairobi, Kenya; 2 University of Nairobi, Nairobi, Kenya; 3 END Fund, New York, New York, United States of America; 4 Ministry of Health, Kigali, Rwanda; George Washington University Medical Center, UNITED STATES OF AMERICA

## Abstract

**Introduction:**

Soil-transmitted helminth (STH) and schistosomiasis (SCH) infections remain some of the most prevalent neglected tropical diseases (NTDs), causing significant morbidity in most of sub-Saharan Africa (SSA), including Rwanda. With dwindling international funding for NTD services and recent commitments focused on other diseases considered easier to eliminate as a public health problem, it is essential to assess domestic financing sources’ scale, efficiency, and effectiveness. The study aims to strengthen domestic efforts towards sustainable financing for neglected tropical disease programs in Africa, particularly in Rwanda.

**Method:**

Up to 235 patients from 24 health centers in four districts of Rwanda were sampled for this survey. The districts selected had the highest number of STH and SCH based on routine data from June 2021 to December 2022, which is the window period of the study. We estimated affordability using the lowest-paid government worker (LPGW) and then compared this with household income and expenditure obtained from patients participating in the survey. Data was collected from August to September 2023. Limited secondary data were collected to complement primary data. Descriptive statistical analysis was used to present the findings.

**Results and Conclusions:**

The most available drugs were mebendazole, with 100% of facilities reporting no stockout. Praziquantel (PZQ) was the most unavailable drug, reporting 92% stockout at the time of the survey, mainly due to delays in getting supplies from MDA-implementing health facilities. Diagnostics for SCH are the most inaccessible lab services. On average, the total cost (both direct and opportunity cost) to access and utilize STH and SCH services was USD 0.72 (RWF 861.92) and USD 0.96 (RWF 1136.41) for male and female patients, respectively. Although the assessment revealed that treatment for STH and SCH was affordable for the LPGW, women pay a 33% higher cost than men to access NTD services. While services are generally satisfactory, the reimbursement processes are slow, hindering timely access and utilization of SCH and STH services at the health facilities in Rwanda.

While the access and utilization of STH and SCH services in health centers are generally promising, the findings underscore the potential for improvement. By addressing the efficiency in the supply of praziquantel drugs and enhancing reimbursement timelines, we can ensure the continuity and effectiveness of these services, offering hope for a brighter future in the fight against neglected tropical diseases.

## Author summary

This study explores how Rwanda is working to sustainably finance healthcare services for two neglected tropical diseases—soil-transmitted helminths (STH) and schistosomiasis (SCH)—as international aid for such diseases declines. The research surveyed 235 patients across 24 health centers in districts most affected by these conditions, focusing on how accessible, affordable, and well-utilized these services are under Rwanda’s domestic health financing mechanisms.

Key findings show that while basic medications like mebendazole are widely available, others like praziquantel face frequent stockouts due to delayed reimbursements from the CBHI to clinics. Women experience longer travel and waiting times, resulting in higher opportunity costs than men. Yet, out-of-pocket expenses remain low—under USD 1 per visit—suggesting minimal financial risk to patients across income brackets.

Patients rated the quality and acceptability of services highly, though most facilities lacked NTD treatment protocols. The study underscores how domestic efforts—such as budget allocations and health insurance coverage—have helped improve access. However, it stresses the need to streamline reimbursement processes and strengthen supply chains to avoid disruptions in drug availability. The findings provide valuable insight for policymakers aiming to reduce dependency on external funding while enhancing equitable access to NTD care.

## Introduction

Health systems across sub-Saharan Africa (SSA) pose significant barriers to the population’s access to health services. There are infrastructural gaps, particularly in rural areas, which experience geographical barriers to health facilities, poor medical supplies, and weak digital health infrastructure [1]. Human resource shortages, urban-biased distribution, and governance challenges continue to pose essential access barriers [2]. Apart from these, perhaps the most significant challenge remains financing, where many countries on the continent are far from meeting their health financing goals, e.g., less than seven countries met the Abuja target, and many countries’ out-of-pocket payments remain unacceptably high with low domestic spending and continued reliance on external resources [3].

With increasing anxiety over the long-term financing from external sources exemplified by the exit of USAID and sustainability challenges, one program area of concern is the financing of neglected tropical diseases (NTDs). NTDs are diverse communicable yet preventable parasitic, viral, and bacterial diseases that affect over 1.0 billion of the world’s poorest and vulnerable populations primarily in tropical and sub‐tropical settings in Africa, Asia, and the Americas [4–6]. Up to 40% of the affected populations live in Africa, with the poorest and most vulnerable residing in hard-to-reach geographical areas [7–9]. Among the most prevalent NTDs, schistosomiasis (SCH) and soil-transmitted helminths (STH), or intestinal worms, remain a global public health concern and are highly prevalent in low and middle-income countries. They primarily affect poor communities with no access to potable water [10]. It is estimated that about 251.4 million people globally required preventive treatment for SCH in 2021, of which 93% of the total burden is found in sub-Saharan Africa [11].

Despite the existence of treatment and preventive measures, NTDs continue to be responsible for many years of life lost due to disability and premature death among the poor [12]. NTDs, therefore, constitute a disproportionate health burden to marginalized populations and exacerbate inequity. The Sustainable Development Goals (SDG 3) seek to close the health equity gap and have highlighted NTDs as necessary in achieving this objective. Improving access to NTD services is paramount to the SDGs agenda of reducing disease burden and improving health outcomes for all. Among children, NTDs contribute to disabilities and cognitive impairment, limiting their ability to progress with education and improve their living standards in adulthood [13]. Among adults, NTDs limit productivity and directly impact economic development [14]. Factors that exacerbate the NTD burden include poverty and marginalization, underfunding for health systems, and poor surveillance data [15,16].

The endemicity, the burden, and poor financing of NTDs in Africa are well documented [6,13,16–21]. Rwanda is considered one of the best-performing countries in Africa in its efforts to improve access to health services through public health insurance schemes and increased government spending on healthcare. For example, while Rwanda’s current health expenditure as a share of GDP stands at about 7.2%, its neighbours such as Kenya, Ethiopia, Uganda and Tanzania average 3% of GDP as of 2020 [22] However, the country remains endemic for two of the five NTDs amenable to preventive chemotherapy (PC) [23]. National prevalence of SCH ranged between 0% and 70% in areas surrounding water bodies in 2008, and the prevalence has declined significantly over the years, according to recent assessment reports [23,24]. In 2020, data yet to be published by the Rwanda Biomedical Centre (RBC) showed that the threshold for elimination as a public health problem was reached in 97% of high-risk villages selected to assess the impact. In contrast, the national prevalence of STH infections stood at 41% as of 2020 [7,23,25]. However, the 2020 impact assessment found that adults remain the most infected compared to school-aged children, i.e., 48% versus 41%.

In light of diminishing international support for NTD programs [26], and to maintain the gains in the fight against NTDs and increase service coverage for all, there is greater emphasis on domestic financing for sustainability. Currently, financing STH, SCH, and other related NTDs in Rwanda heavily depends on external sources. From the approved budgets, the funding for STH and SCH was RWF 6,250.59 million (USD 6.16 million) in the financial year 2021/22 and RWF 9048.54 million (USD 8.46 million) in 2022/23. External sources accounted for almost 92% of overall STH and SCH spending RWF 14,032.71 (USD 13.47 million) in the FYs 2021/22 and 2022/23. However, the government contribution significantly increased from RWF 126.33 (USD 0.12) million in 2021/22 to RWF 1100.388 (USD 1.07) million in 2022/23.

Despite the increase in government budget, external funding remains significant and could grossly affect NTD service delivery if abruptly cut. To reduce dependency on external funding for NTDs, the government aims to increase budget allocation to account for at least 40% of the NTD budget to ensure sustainability [27]. Alongside budget increases, the Rwandan Government has improved access to NTD services by rolling out bi-annual mass drug administration (MDA), ensuring that over 65 million treatments against intestinal worms and schistosomiasis were delivered to children aged 1–15 years since 2008 [28]. The government has taken further steps to improve access to NTD treatment and preventive services by progressively integrating NTDs into the national health insurance benefit package- the Community-Based Health Insurance (CBHI) scheme.

Rwanda’s CBHI scheme was initiated by the Rwandan Government in 2005 to spread the financial burden of medical costs and ensure that the poor and vulnerable households have access to essential health services without incurring a financial burden [29]. The government subsidizes the CBHI scheme at almost 50% of the total funding [29–31]. The benefits package includes comprehensive preventive and curative services and essential drugs at health centers and referral hospitals. Integrating NTDs into the routine health services delivery system is a crucial indicator for domestic financing and is considered a key step toward sustainable NTD programming in endemic countries [32].

While the efforts to eliminate NTDs through sustainable financing in Rwanda are commendable, there are empirical gaps on whether the current funding from domestic sources (government and public insurance) has improved NTD service availability, affordability, and optimized utilization by the population to contribute toward sustainable elimination. For instance, STH and SCH services are expected to be available and affordable to all those in need of the services, but the range of services covered, their availability, demand, and utilization, and who bears the cost burden in trying to access SCH and STH services, is not well-understood in Rwanda. The aim of this study is, therefore, to provide evidence on the implications of public financing of NTDs on service access. This will assist the government and the CBHI in improving domestic resource mobilization and allocation for effective and sustainable NTD elimination.

## Methodology

### Ethics statement

A protocol was submitted to the Ethics and Scientific Review Committee of the Rwanda National Research Ethics Committee (RNEC) and the Rwanda Biomedical Centre (RBC). Approval for the study was received from RNEC (RNEC-IRB 0001497 of I0RGOOO1100) on August 11, 2023—collaboration letter from the Rwanda Biomedical Centre (RBC), Ref. No M84/RBC/2023, provided a human subjects exemption for this research on 13 October 2023.

### Design and setting

The study relied mainly on primary survey data collected through exit interviews. Secondary data were collected to inform the NTD financing situation in Rwanda and to understand the government budget for operational costs related to mass drug administration (MDA).

This study was conducted in 24 randomly selected health centers in four Rwanda districts: Gisagara, Rubavu, Burera, and Rutsiro. These are amongst the districts with the co-endemicity for STH and Schistosomiasis as reported in the Rwanda NTDs Strategic Plan 2019–2024 [24]. In addition, these districts had a high number of STH and SCH, all combined from June 2021 to December 2022, which is the window period of the study. All the selected health centers were providing STH and SCH services (deworming medications including albendazole, mebendazole, and praziquantel; diagnostics for Schistosomiasis (SCH) and STH or intestinal worms) covered by the CBHI scheme.

### Sampling and data collection

The sample size was calculated using the SCH and STH facility visits data for 2022, which was estimated at 207,572 visits. Assuming each of the 24 facilities had an equal number of visits during the year 2022, each facility received about 8649 SCH and STH patients, which translates to about 24 visits per day in each facility. Aiming for a higher sample size, the study chose to interview 50% of the census sample, i.e., every second patient in each facility was interviewed, which gave a total sample size of 288 SCH and STH patients. 235 patients were accepted to participate in the study, with an acceptable response rate of about 82%. In addition, 24 facility in-charges from the 24 health facilities were interviewed to assess stock levels of SCH and STH drugs.

A total of four data collectors were assigned responsibility for collecting data at each of the 24 facilities. Each two-person team split responsibility between collecting facility data of individual facilities and interviewing individual study participants to assess the availability, affordability, and utilization of STH and SCH services. Data collection was completed between August and September 2023. Exit interview participants were recruited from the 24 facilities among the target populations in need of STH and SCH services who came to a study site seeking these services. Individual participants were selected after they had received STH and SCH services at the respective facility. The research assistants explained the study to each potential participant, using an approved recruitment script, and all respondents were asked if they were willing to voluntarily participate in the study. All willing participants signed a consent form after acknowledging their understanding of the purpose of the study. Exit interviews were conducted with each eligible participant based on their willingness to participate. The information collected included demographic characteristics of the respondents (age, religion, membership in a health insurance scheme, marital status, individual income, household income, level of education, employment status), transport costs, amount paid for drugs, travel time to and from the facility, and waiting time. English and Kinyarwanda translations of the participant and facility questionnaires were loaded onto a mobile data collection application, *KoBoCollect*, which was used to record responses.

In this study, we considered three key dimensions of access [33] which include: (i) availability of services (drugs and non-drug pharmaceuticals, physical accessibility including distance to facility and travel time, and availability of qualified staff (ii) affordability of services including cost of medicine, travel costs, cost of consultation incurred concerning tests/investigations, other indirect costs); (iii) acceptability of services such as social and cultural context (gender) affecting accessibility, and individual patient perceptions of quality of services.

Accessibility was assessed using the WHO availability target of greater than or equal to 80%. Affordability was estimated based on the average monthly wage for the lowest-paid government worker (LPGW). From an economic costing perspective, affordability was also estimated using direct financial expenses and opportunity costs. Costs to STH and SCH patients are largely restricted to the charges for the transport costs incurred from home to the treatment site and opportunity costs. The out-of-pocket costs included round-trip transportation costs, which were assessed through the exit interviews, and the time required for obtaining treatment, which included the round-trip travel time to the facility, waiting time, and time for consultation until exit from the facility. The patient’s daily earnings give an estimate of the value of time spent at the health facility.

### Statistical analyses

Data were analyzed using STATA version 17. Simple descriptive statistics assessed the availability, affordability, and utilization/demand of the selected STH and SCH drugs and related services. An availability of 80% or higher was used as the benchmark for accessibility as per WHO guidelines [34]. The combined availability of commodities that were surveyed was calculated to provide the overall availability of that specific commodity at the facility. Stockout information was sought from all the selected STH and SCH commodities covered by the CBHI scheme, which were supposed to be available at that level of care, regardless of whether they were in or out of stock at the time of the survey. Stockout was calculated as the percentage of facilities that reported at least one stockout of the selected STH and SCH commodity over the measured period, with stockout days calculated as the average number of days stockouts of a commodity lasted per facility.

To calculate affordability, the median price of the entire treatment course of a commodity was compared to the official salary of the lowest-paid government worker (LPGW), per day in 2023. If a commodity price exceeded one day’s wages, it would be considered unaffordable based on the World Bank’s international poverty line (IPL) of USD 2.15 per person per day. Accessibility was calculated using the availability and LPGW affordability measures. This resulted in a composite measure in which accessibility is achieved with an 80% or higher availability and a price of less than a day’s wage for an LPGW. The results were then compared with information on income and expenditure obtained from patients participating in the survey.

## Results

### Socio-demographic characteristics

The results, presented in Table 1, revealed that the respondents tended to be young, with 39.2% under 30 years old. About the same number of respondents were single and never married (28%) or were living together (28.1%).

**Table 1:** Descriptive characteristics of the full sample.

Variable		Mean/%
**District**	Rubavu	25.5%
	Rutsiro	26%
	Gisagara	23.4%
	Burera	25.1%
**Gender**	Male	37%
	Female	63%
**Age**	18-20	12.8%
	21-25	16.6%
	26-30	9.8%
	31-35	10.2%
	36-40	10.2%
	41-45	10.6%
	46-50	9.8%
	51-55	5.5%
	56+	14.5%
**Marital Status**	Living Together	28.1%
	Married/In-Union	23.4%
	Never Married/Single	28.1%
	Widowed/Separated/Divorced	20.4%
**Education**	No grade completed	17%
	Primary incomplete	25.9%
	Primary complete	30.2%
	Secondary incomplete	17%
	Secondary complete	9.4%
	Tertiary/college complete	0.4%
**Religion**	Muslim	3.4%
	No Religion	3.8%
	Protestant/ Other Christian	37.9%
	Roman Catholic	54.9%
**Currently employed**	Yes	68%
	No	32%
**Insurance cover**	Yes	99.2%
	No	0.8%

### Availability and range of STH and SCH services

**Fig 1:**
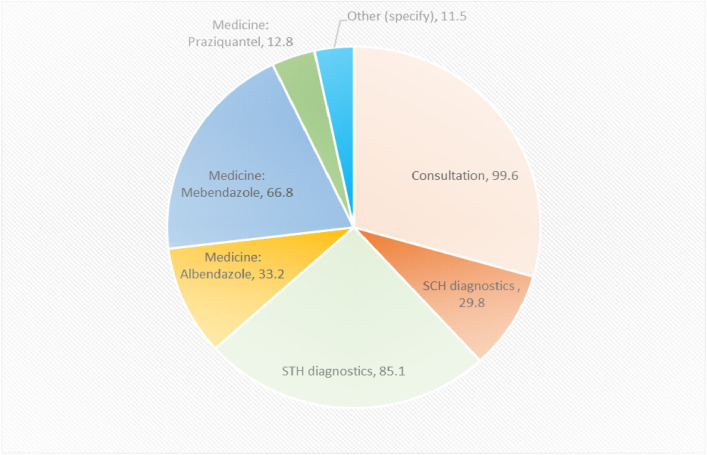
NTD services received during visits to health facilities.

As depicted in Fig 1, almost all the patients (99%) received consultation services, 29.8% received SCH diagnostic services, and 85% received STH diagnostics. A third of the respondents (33%) received the prescribed albendazole medicine, and 66.8% and 12.8% received the prescribed mebendazole and praziquantel medicines.

**Fig 2:**
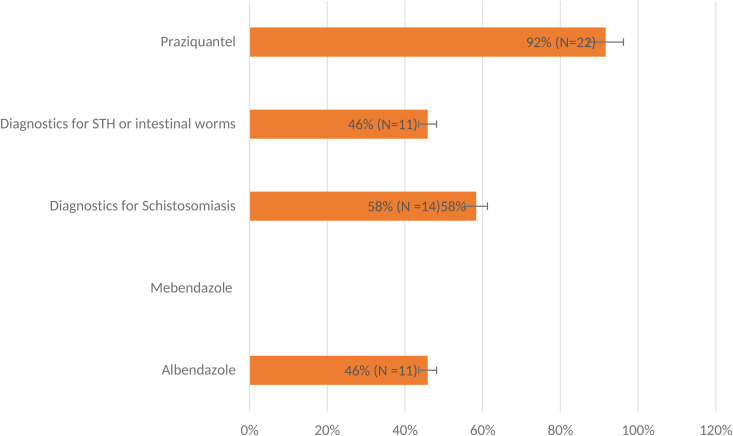
Proportion of facilities with stockout of STH/SCH drugs, FY2022/23.

Regarding stockouts of STH and SCH drugs on the day of the survey (Fig 2), 22 (92%) out of the 24 facilities did not have praziquantel drugs, and 11 (46%) had no diagnostics for STH or intestinal worms. In comparison, diagnostics for schistosomiasis were unavailable in 14 (58%) of the facilities. However, mebendazole drugs were available in all 24 health facilities. Note that, as the primary strategy for the NTD elimination programs, preventive chemotherapy (PC) employs anthelmintics, such as albendazole (ADZ) or mebendazole (MBDZ) for STHs, ADZ and diethylcarbamazine (DEC) and/or ivermectin (IVM) for LF and onchocerciasis, and praziquantel (PZQT) for SCH [35].

The stockout of STH/SCH drugs forced patients to either use an alternative drug, e.g., mebendazole in place of albendazole, or to visit the facility another day when supplies were available. Alternatively, those who could afford it were advised by health workers to purchase at private facilities.

As noted by the study, one of the major contributors to drug stockouts is a delay in receiving reimbursements for services provided. At the time of the survey, 23 out of the 24 facilities in the sample indicated that they experienced delays in receiving reimbursements from the CBHI through the Rwanda Social Security Board (RSSB) for services provided during FY 2021/22 and FY 2022/23. Table 2 presents the estimated number of months of delays in receiving the funds.

**Table 2:** Number of months of delay in receiving funds/reimbursements from the CBHI scheme.

Duration it took to have funds transferred to the facility	Freq.	%
Between 2–4 months	18	75
Over a month	6	25
Total	24	100

About 75% of the facilities experienced 2–4 months of delays. The respondents cited several causes for the delays, including errors in preparing the invoice (37.5%), delays in submitting the invoice by the facilities (45.8%), delayed reimbursement from the CBHI scheme (79.2%), and delays in verifying the invoices (20.8%). The delayed reimbursement does not affect SCH and STH services alone, but the availability of all services is affected since health facilities cannot replenish their entire stocks before funds are made available.

The study also considered the time taken to receive services (travel and waiting time) as a measure of availability in the timely use of NTD services. On average, women spent more time than men receiving NTD services (307.4 minutes or 5.0 hours for women compared to 238.5 minutes or 4.0 hours for men). In travel time, women spent about 95.93 minutes (approximately 2 hours) traveling to health facilities versus 78.94 minutes (approximately 1.3 hours) spent by men. Similarly, average time spent with the clinician, average waiting time, and average time after consultation to exit the facility were approximately 159.6 minutes (approximately 2.6 hours) for male patients and 211.5 minutes (3.5 hours) for female patients (Table 3).

**Table 3:** Time taken to receive STH/SCH care across facilities.

Participant category	Av. Travel (mins)	Av. waiting time (mins)	Av. Time from consultation to exit (mins)	Av. Time with a clinician (mins)	Av. cost of Transport
Male	78.94	51.75	91.91	15.94	USD 1.65 (RWF 1911.44)
Female	95.93	71.04	132.82	7.64	USD 1,42 (RWF 1645.00)

There was no immediate explanation for the differences between men and women in the time taken to receive services. However, less travel time and higher transport costs for men compared to women indicate that more men than women used motorbikes or other faster and more expensive means of transportation to the health facility. This suggests an economic advantage for men in accessing health facilities.

### Affordability of SCH and STH services

A summary of patients’ indirect costs (cost of travel and waiting time), direct costs, and total costs for accessing STH and SCH treatment is presented in Table 4. The average total cost (including both direct and opportunity costs) to access and utilize STH and SCH services was US$0.72 (RWF 834.08) and US$0.96 (RWF 1,112.11) for male and female respondents, respectively. The average total cost for the opportunity costs accounted for 1.5% (US$0.45 or RWF 521.30) of the total average cost per day for male respondents. In comparison, the opportunity cost for female respondents accounted for 2.4% (US$0.68 or RWF 787.75) of the total cost per day. The out-of-pocket costs for transportation fares amounted to $0.09 (RWF 104.26) and $0.06 (RWF 69.51) for male and female respondents, respectively.

**Table 4:** Average total costs per client per day.

Patient	Avg. total time (mins)	Cost of time	Avg. travel fare	Cost of medication	Avg. total cost (per trip)
Male	238.5	US$0.45 (RWF 521.30)	US$0.09 (RWF 104.26)	US$0.18 (RWF 208.52)	US$0.72 (RWF 834.08)
Female	305.5	US$0.68 (RWF 787.75)	US$ 0.06 (RWF 69.51)	US$ 0.22 (RWF 254.86)	US$0.96 (RWF 1,112.11)

The findings indicate that the average daily income was highest among male respondents at RWF1,784.01 (US$1.54) against RWF 1,702.92 (US$1.47) for females. The results show disparity in the costs for male and female respondents, which is largely driven by the amount of travel time and, hence, the cost per round trip. Table 5 presents the total average cost of time for the daily visits.

**Table 5:** Average costs of STH and SCH services vs average monthly household income.

Patient category	Av. Monthly income	Medication	Av. Travel fare	Direct costs	Av. Income per day	Av. Total time (mins)	Cost of time (indirect)	Av. Total cost
Male	US$29.37 (RWF 34,023.68)	US$0.18 (RWF 208.52)	US$0.09 (RWF 104.26)	US$0.28 (RWF 324.37)	US$1.54 (RWF 1784.01)	238.5	US$0.45 (RWF 521.30)	US$0.72 (RWF 834.08)
Female	US$28.30 (RWF 32,784.14)	US$0.22 (RWF 254.86)	US$0.06 (RWF 69.51)	US$0.28 (RWF 324.37)	US$1.47 (RWF 1702.92)	305.5	US$0.68 (RWF 787.75)	US$0.96 (RWF 1112.11)

The total cost to patients accessing STH and SCH services for a month was compared with the average monthly household income (Table 5).

**Fig 3:**
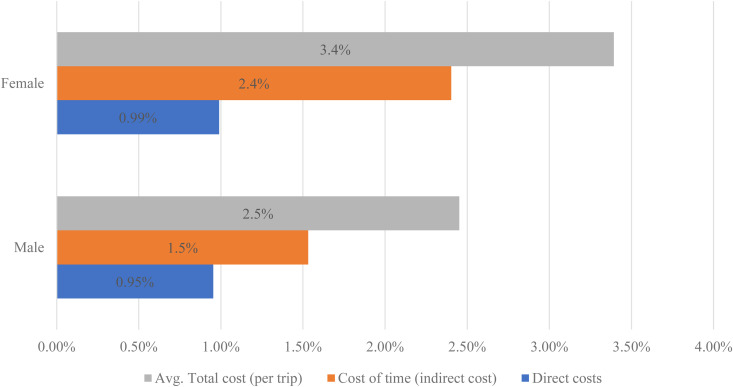
Monthly patient costs as a % of monthly household income.

The results show that male patients paid direct costs of nearly US$0.28 (about 0.95% of average household income per month) for a month (assuming one visit to the health facility per month) to access treatment services at the facilities. The share of direct costs paid by female patients was US$0.28, which is about 0.99% of the average household income per month of US$28.30. The direct costs combined with indirect costs produced a total mean cost burden of US$0.72 or 3.4% of household income per month for male patients and a mean total cost per month of US$0.96 or 2.5% of average monthly household income for female patients (Fig 3).

The total direct and indirect costs as a share of household income are presented in Table 6.

**Table 6:** Total direct and indirect costs as a % of household income.

Household income bracket	Percentile	Household monthly income in $ & RWF	Total cost	Total cost % monthly household income
0-6000	10%	$5.19 (RWF 6000)	$0.17 (RWF 200)	3.33
6001-10000	20%	$8.65 (RWF 10,000)	$0.19 (RWF 220)	2.20
10001-15000	30%	$12.98 (RWF 15,000)	$0.22 (RWF 250)	1.67
15001-19840	40%	$17.16 (RWF 19840)	$0.34 (RWF 391.20)	1.97
19841-23000	50%	$19.90 (RWF 23,000)	$0.43 (RWF 496.87)	2.16
23001-43000	60%	$25.95 (RWF 30,000)	$0.59 (RWF 685.67)	2.29
43001-60000	70%	$37.20 (RWF 43,000)	$0.88 (RWF 1015.63)	2.36
60001-100000	80%	$51.91 (RWF 60,000)	$1.29 (RWF 1488.89)	2.48
100001-250000	90%	$86.51 (RWF 100,000)	$2.02 (RWF 2340.63)	2.34
250000+	100%	$216.28(RWF 250,000)	$10.05 (RWF 11616.67)	4.65

The results show that the poorest households spent about 3.3% of their income on STH and SCH care. On the other hand, those in the middle—and upper-income brackets spent between 1.7% and 2.5% of their income on STH and SCH care and treatment. The wealthiest households with an income of RWF 250,000 spent 4.7% on STH and SCH treatment and care. These findings, including on the expenditures of LPGW, show no real financial risk involving catastrophic payments for STH and SCH services. These findings show that the cost burden of SCH and STH services is progressive because of government subsidies that target low-income earners. Higher income brackets are also likely to spend more on faster means of transportation and also pay out of pocket during stockouts.

Costs by district show Rubavu District is the most expensive for those seeking SCH and STH services. Women incur the highest cost averaging about USD 1.37 against men who incur USD 0.83 per trip (Table 7).

**Table 7:** Variation in mean direct and indirect costs of accessing STH and SCH services by study district.

	Avg. Total time (mins)	Cost of time	Avg. travel fare	Cost of medication	Avg. Total cost (per trip)
Male	238.5	$0.46 (RWF 529.57)	$0.10 (RWF 112.64)	$ 0.19 (RWF 219.71)	$ 0.75 (RWF 861.92)
Female	305.5	$0.70 (RWF 808.22)	$ 0.06 (RWF 68.24)	$ 0.22 (RWF 259.95)	$ 0.98 (RWF 1136.41)
BURERA DISTRICT					
	**Avg. Total time (mins)**	**Cost of time**	**Avg. travel fare**	**Cost of medication**	**Avg. Total cost (per trip)**
Male	169.35	$ 0.40 (RWF 460.79)	$ 0.29 (RWF 339.13)	$ 0.18 (RWF207.61)	$ 0.87 (RWF 1007.53)
Female	297.64	$ 0.56 (RWF 641.72)	$ 0.14 (RWF 166.67)	$ 0.30 (RWF 346.11)	$ 1.00 (RWF 1154.5)
GISAGARA DISTRICT					
	**Avg. Total time (mins)**	**Cost of time**	**Avg. travel fare**		**Avg. Total cost (per trip)**
Male	248.46	$ 0.16 (RWF 186.04)	$0.00	$ 0.19 (RWF 218.46)	$ 0.35 (RWF 404.5)
Female	310.71	$ 0.53 (RWF 608.35)	$0.00	$ 0.17 (RWF 192.14)	US$ 0.69 (RWF 800.5)
RUBAVU DISTRICT					
	**Avg. Total time (mins)**	**Cost of time**	**Avg. travel fare**	**Cost of medication**	**Avg. Total cost (per trip)**
Male	297.91	$ 0.60 (RWF 697.36)	US$0.08	$ 0.18 (RWF 204.55)	$ 0.86 (RWF 992.82)
Female	344.89	$ 1.17 (RWF 1356.5)	US$0.06	$ 0.19 (RWF 215.58)	$ 1.42 (RWF 1640.5)
	**Avg. Total time (mins)**	**Cost of time**	**Avg. travel fare**		**Avg. Total cost (per trip)**
Male	243.93	$ 0.53 (RWF 610.82)	$0.00	$ 0.21 (RWF 241.38)	$ 0.74 (RWF 852.2)
Female	260.81	$ 0.52 (RWF 606.78)	$ 0.04 (RWF 46.88)	$ 0.26 (RWF 304.69)	US$ 0.83 (RWF 958.34)

### Acceptability of SCH/STH services

In addition to cost, the quality of services is an important determinant of using STH and SCH services. In this study, respondents were asked to record their level of satisfaction concerning: (i) quality of treatment received, ii) privacy and confidentiality, iii) timeliness in the provision of services, (iv) facility cleanliness and hygiene, (v) attitude towards patients, (vi) overall rating. A rating scale (excellent, good, fair, and poor) was used. Most respondents (78.3%) rated the services provided as good, while 17.5% rated them as fair and 1.7% rated the services as poor. Other aspects of acceptability (e.g., cleanliness and hygiene, privacy of treatment and waiting time, and staff attitude) were similar, indicating that an overwhelming majority of the respondents viewed the services as acceptable (Table 8).

**Table 8:** Satisfaction with SCH and STH services at health facilities.

Type of services	Rating of services (%)				Total (N)
	Excellent	Good	Fair	Poor	
Quality of service	2.6 (6)	78.3 (184)	17.5 (41)	1.7 (4)	235
Privacy/confidentiality	7.7 (18)	73.2 (172)	19.2 (45)	0.0	235
Timeliness of service provision	7.2 (17)	68.1 (160)	19.6 (46)	5.1 (12)	235
Cleanliness of the facility	1.7 (4)	86.4 (203)	10.2 (24)	1.7 (4)	235
Attitude towards patients	0.85 (2)	80.9 (190)	17.9 (42)	0.4 (1)	235
Overall rating	2,6 (6)	95.3 (224)	0.0	2.1 (5)	235

In addition, the study assessed the availability of treatment protocols as a measure of improved patient-centered outcomes. The survey results revealed that 21 (88%) of the facilities did not have NTD treatment protocols for various NTDs, and only three facilities reported availability of treatment protocols for NTDs.

### STH and SCH service demand and utilization

The utilization of STH and SCH services decreased slightly from 215,267 OPD visits in 2021–207,572 OPD visits in 2022 (Table 9).

**Table 9:** Annual visits for STH/SCH services covered by CBHI scheme.

Intervention	2021	2022	Total	% Total
Intestinal worm medication (albendazole & mebendazole)	177,492	170,457	347,949	82.3%
Diagnostics for Schistosomiasis (SCH)	1,732	136	1,868	0.4%
Diagnostics for STH or intestinal worms	36,043	36,979	73,022	17.3%
**Total**	**215,267**	**207,572**	**422,839**	**100.0%**

The data show that most STH and SCH patients were treated asymptomatically. Patients receiving intestinal worm medications (mebendazole or albendazole) accounted for 82.3% of the total outpatient (OPD) visits over the two years. In contrast, diagnostics accounted for 17.3% of the visits for STH and 4% for SCH.

In terms of utilization by district, the health facilities in Burera district accounted for 29.1% and 29.7%, respectively, of the total utilization of STH and SCH services, followed by Rubavu, which accounted for 26.7% and 26.2% in 2021 and 2022, respectively. Facilities in the Gisagara district reported the lowest utilization, at 17.7% in 2021 and 18.8% in 2022 (Table 10).

**Table 10:** Comparison of utilisation of STH and SCH services by district.

District	2021	%	2022	%
Rubavu	57,462	26.7%	54,294	26.2%
Gisagara	38,140	17.7%	39,124	18.8%
Rutsiro	57,098	26.5%	52,479	25.3%
Burera	62,567	29.1%	61,675	29.7%
**Total**	**215,267**	**100.0%**	**207,572**	**100.0%**

The number of OPD visits for all STH and SCH services during the two years declined slightly, except for intestinal worm medication. The largest drop in outpatient utilization (OPD) was observed in the diagnostics for Schistosomiasis (from 1732 OPD visits in 2021–136 OPD visits in 2022), followed by patients for deworming medications (mebendazole), whose utilization declined from 79,233 in 2021–70,826.

## Discussion

There are concerns about access and sustainability of health services in general and NTD programs in particular due to overreliance on external funding and low domestic investments. Sustainability means the capacity of a health system to maintain the gains toward NTD prevention and control on the path to elimination. Poor access to NTD services has broader policy implications beyond the health system. It imposes an economic burden by reducing productivity and contributes to a cycle of poverty and social exclusion [36]. Late-stage presentations of most NTDs are not just fatal but are a significant burden to health facilities and jeopardize progress toward Universal Health Coverage. The United Nations SDGs recognize universal access to NTD services as integral to achieving UHC [37]. On the other hand, children afflicted with NTDs have lower educational achievement as NTDs increase absenteeism and cognitive impairment, therefore potentially contributing to lower social status [13,38].

As a largely neglected program in many low- and middle-income countries, countries are encouraged to invest domestic resources to control and eliminate NTDs. In Rwanda, the bulk (90%) of funding for STH and SCH prevention and elimination is external. However, the Rwanda Government has progressively relied less on external support to control and eliminate NTDs. The steps include (i) the introduction of a budget line for procurement of NTD drugs, (ii) the design and delivery of a package of NTD care under the CBHI scheme, (iii) the adoption of one health policy that commits to enhance collaboration of different actors of NTD stakeholders, (iv) budget allocation for MDA operational costs, and (v) decentralization of NTD interventions and integration of NTD prevention efforts to the village level under district coordination. The study focused on understanding how public domestic financing through the Rwandan government’s introduction of a budget line for NTD drugs and financing under the CBHI scheme has influenced access to and utilization of SCH and STH services in Rwanda. The introduction of a budget vote influences access to healthcare by guaranteeing a resource basket, affecting the funding available for health services [39]. In so doing, it contributes to financial risk protection by lowering the cost of care for individuals.

There is a growing body of literature on NTD service access (availability, affordability, and utilization) with mixed results [16,40–42]; e.g., in Ethiopia, Kassahun et al. [40] showed that service availability is relatively good with up to 64% of all health facilities offering STH services, but only 40% had SCH services. Our findings show a similar trajectory of inconsistency in the availability of SCH and STH drugs; e.g., praziquantel is the least available medicine with a stockout rate of 92% because of delays in receiving supplies from MDA implementing health facilities. This is followed by albendazole, received by just 33% of patients seeking care. On the other hand, mebendazole was the most available drug with 100% facilities stocked. In other NTDs, Ooms et al. [41] report low availability of snakebite commodities (43%) in public health facilities. Similarly, the Ethiopian Public Health Institute [43] found that the availability of drugs for NTD treatment varied by facility type, with hospitals more likely than lower-level facilities to be stocked. One of the most cited causes of stockouts of essential drugs is delayed reimbursements to health facilities to allow for timely re-stocking of commodities [44,45]. The consequences of a lack of availability of essential NTD services are not only costly to those seeking care but also discourage repeat visits, which constitutes a barrier to NTD control and elimination. The reimbursements to facilities need to be predictable and the supply chain strengthened, to ensure consistency in the availability of critical NTD services, particularly those prepaid through publicly financed institutions.

The availability of SCH and STH services is hampered by distance to health facilities, with women disproportionately affected. Of the 85% of the respondents who walked to a health facility, women spent on average 96 minutes compared to 79 minutes spent by men. The extra time spent by women traveling to health facilities not only has cost implications but also constitutes a physical barrier to accessing NTD services. Although the government took steps to reduce walking time to facilities by 50% (from 95 minutes to 47 minutes) in 2020 [46], the evidence suggests that there are still significant challenges in accessing health facilities in some districts. The steps taken by the government include the construction of health posts and the deployment of community health workers to bring services closer to communities [46]. The use of CHWs to bring NTD services closer to the communities has been proven effective in malaria control and improving maternal and child health services [47,48]. It is highly recommended for effective and sustainable control and elimination of NTDs.

What remains encouraging in Rwanda is that SCH and STH services remain essentially affordable with no evidence of catastrophic expenses. Usually, out-of-pocket health expenditure is considered catastrophic and impoverishing when a household is forced to cut down on subsistence needs, sell productive assets, incur debts, or utilize savings to meet health needs [34,41,49,50]. Similar to this study, Dong et. al. [51] reported better affordability overall but also noted a large gap between the affordability levels of originator brands (OBs) and lowest-priced generics (LPGs) of essential drugs in Ethiopia.

The affordability of SCH and STH services in Rwanda was primarily influenced by travel and waiting time, whose costs were higher than the expenses for the actual NTD services at health facilities. Noting that the indirect medical expenses disproportionately affected women calls for considering the driving factors in the analysis of access to NTD services in a low-resource setting, such as Rwanda. Women spent about a dollar more than men to access health facilities- a cost mainly attributed to more extended travel and waiting time, which are likely to be an important determinant of STH and SCH services utilization. Saxena et al. [52] have reported cost-related disparities between men and women in access to healthcare.

Affordability is a critical determinant of access to health services in most settings, particularly in low- and middle-income countries (LMICs). Generally, essential health services are found to be relatively unaffordable to many people [16,42,49,53–55]. However, treatment costs for STH and SCH services are affordable in a low-income setting, requiring only 13% of a day’s wages from the LPGW. This suggests that NTD control and prevention is one of the best buys in public health available to those affected community members. Studies such as Ooms et. al. [40] have also demonstrated the affordability of NTD services, excluding snake anti-venoms, which are found to be unaffordable to most households in Kenya. Governments should, therefore, be encouraged to invest more in NTD control and elimination as a less costly alternative to the continued neglect of NTDs. Rwanda currently funds operational costs of MDAs, which indicates that significant costs can be domestically resourced when appropriately prioritized through political commitment, effective advocacy, governance, multisectoral coordination structures, and integration within the broader health system budgeting and planning processes.

Evidence of steady demand and utilization of STH and SCH services in Rwanda indicates efficiency in the use of investments in NTDs, wherein the target population utilizes the services purchased; i.e., the population’s NTD needs are well aligned with the services purchased. There is also a clear linkage between service use and the reported high patient satisfaction with the services offered. However, the demand and utilization can still be improved with timely reimbursements to health facilities and a strengthened procurement and supply chain for NTD commodities. Satisfaction with health services is a contentious issue, especially in LMIC, with many reporting poor services associated mainly with poor staff attitude, ineffective physician consultations, and inefficient admission process [52,53]. However, in Ghana, there was an overwhelming majority (>95%) of patients who had a positive perception of the quality of services [19]. Our study reported 95.3% satisfaction with SCH and STH services, which means that, although the quality of services is generally low in low-resource settings, there is evidence from both the Ghanaian and Rwandan experiences that this can change for the better.

The WHO emphasizes that the goals of UHC cannot be effectively achieved if the quality of services is not satisfactory. The surest way to sustain and eliminate neglected diseases is to combine adequate domestic financing with improved access to and utilization of quality NTD services. This combination needs to be approached from an equity lens, considering the identified gender gaps in access to SCH and STH services.

## Study limitations and potential biases

The study is aware of biases related to self-reported data and ensured that the data collected from individual participants were triangulated with discussions with health workers. Micro-level gaps, such as socioeconomic indicators and behavioral determinants of health, were either not relevant to the objective of the study or addressed by having well-trained interviewers to ensure data consistency. The generalizability concerns have been addressed through triangulated data sources and a non-biased sampling process of 24 primary health facilities in different districts representing urban, peri-urban, and rural areas. However, the study acknowledges that the small sample size focusing on primary health facilities may limit generalization in hospital settings.

## References

[1] Oleribe OO, Momoh J, Uzochukwu BS, Mbofana F, Adebiyi A, Barbera T, et al. Identifying key challenges facing healthcare systems in Africa and potential solutions. International Journal of General Medicine. 2019;12:395–403. 31819592 10.2147/IJGM.S223882 PMC6844097

[2] WHO. Health systems in Africa: community perceptions and perspectives. 2012. https://www.afro.who.int/sites/default/files/2017-06/english---health_systems_in_africa---2012.pdf

[3] WHO. State of health financing in the African region. 2013. https://www.afro.who.int/sites/default/files/2017-06/state-of-health-financing-afro.pdf

[4] Hotez PJ, Kamath A. Neglected tropical diseases in sub-saharan Africa: review of their prevalence, distribution, and disease burden. PLoS Negl Trop Dis. 2009;3(8):e412. doi: 10.1371/journal.pntd.0000412 19707588 PMC2727001

[5] Mitra AK, Mawson AR. Neglected tropical diseases: epidemiology and global burden. Trop Med Infect Dis. 2017;2(3). 10.3390/tropicalmed2030036 PMC6082091 30270893

[6] WHO. Neglected tropical diseases. Geneva: World Health Organization. 2018.

[7] Rujeni N, Morona D, Ruberanziza E, Mazigo HD. Schistosomiasis and soil-transmitted helminthiasis in Rwanda: an update on their epidemiology and control. Infect Dis Poverty. 2017;6(1):8. doi: 10.1186/s40249-016-0212-z 28245883 PMC5331630

[8] Uniting to Combat NTDs. Powering our partnership to fight all 20 neglected tropical diseases. 2018. http://unitingtocombatntds.org/resource/London-declaration

[9] WHO. Countries NTD masterplan 2021 - 2025 framework for development. Brazzaville: World Health Organization office for African Region. 2020.

[10] GBD 10, Disease and injury incidence and prevalence collaborators. Global, regional and national incidence, prevalence and years living with disability for 354 diseases and injuries for 195 countries and territories, 1990-2017: a systematic analysis for the Global Burden of Disease Study 2017. Lancet. 2017;392(10159):1789–858. 10.1016/S0140-6736(18)32279-7 PMC6227754 30496104

[11] Adenowo FA, Oyinloye BE, Ogunyinka BI, Kappo AP. Impact of human schistosomiasis in sub-Saharan Africa. Braz J Infect Dis. 2015;19(2):196–205. 25636189 10.1016/j.bjid.2014.11.004 PMC9425372

[12] de Silva NR, Brooker S, Hotez PJ, Montresor A, Engels D, Savioli L. Soil-transmitted helminth infections: updating the global picture. Trends Parasitol. 2003;19(12):547–51. doi: 10.1016/j.pt.2003.10.002 14642761

[13] Ochola EA, Karanja DMS, Elliott SJ. The impact of Neglected Tropical Diseases (NTDs) on health and wellbeing in sub-Saharan Africa (SSA): A case study of Kenya. PLoS Negl Trop Dis. 2021;15(2):e0009131. doi: 10.1371/journal.pntd.0009131 33571200 PMC7904142

[14] Lenk EJ, Redekop WK, Luyendijk M, Rijnsburger AJ, Severens JL. Productivity Loss Related to Neglected Tropical Diseases Eligible for Preventive Chemotherapy: A Systematic Literature Review. PLoS Negl Trop Dis. 2016;10(2):e0004397. doi: 10.1371/journal.pntd.0004397 26890487 PMC4758606

[15] Johnston EA, Teague J, Graham JP. Challenges and opportunities associated with neglected tropical disease and water, sanitation and hygiene intersectoral integration programs. BMC Public Health. 2015;15:547. doi: 10.1186/s12889-015-1838-7 26062691 PMC4464235

[16] Ukwaja KN, Alphonsus C, Eze CC, Lehman L, Ekeke N, Nwafor CC, et al. Investigating barriers and challenges to the integrated management of neglected tropical skin diseases in an endemic setting in Nigeria. PLoS Negl Trop Dis. 2020;14(4):e0008248. doi: 10.1371/journal.pntd.0008248 32352967 PMC7217480

[17] de Vlas SJ, Stolk WA, le Rutte EA, Hontelez JAC, Bakker R, Blok DJ, et al. Concerted Efforts to Control or Eliminate Neglected Tropical Diseases: How Much Health Will Be Gained?. PLoS Negl Trop Dis. 2016;10(2):e0004386. doi: 10.1371/journal.pntd.0004386 26890362 PMC4758649

[18] Hotez PJ. One world health: neglected tropical diseases in a flat world. PLoS Negl Trop Dis. 2009;3(4):e405. doi: 10.1371/journal.pntd.0000405 19399165 PMC2668801

[19] Hotez PJ, Fenwick A, Savioli L, Molyneux DH. Rescuing the bottom billion through control of neglected tropical diseases. Lancet. 2009;373(9674):1570–5. doi: 10.1016/S0140-6736(09)60233-6 19410718

[20] Houweling TAJ, Karim-Kos HE, Kulik MC, Stolk WA, Haagsma JA, Lenk EJ, et al. Socioeconomic Inequalities in Neglected Tropical Diseases: A Systematic Review. PLoS Negl Trop Dis. 2016;10(5):e0004546. doi: 10.1371/journal.pntd.0004546 27171166 PMC4865383

[21] Ochola EA, Elliott SJ, Karanja DMS. The Impact of Neglected Tropical Diseases (NTDs) on Women’s Health and Wellbeing in Sub-Saharan Africa (SSA): A Case Study of Kenya. Int J Environ Res Public Health. 2021;18(4):2180. doi: 10.3390/ijerph18042180 33672237 PMC7926948

[22] WHO. WHO African Region Health Expenditure Atlas 2023. Brazzaville: WHO African Region. 2024.

[23] Ruberanziza E, Owada K, Clark NJ, Umulisa I, Ortu G, Lancaster W, et al. Mapping Soil-Transmitted Helminth Parasite Infection in Rwanda: Estimating Endemicity and Identifying At-Risk Populations. Trop Med Infect Dis. 2019;4(2). 10.3390/tropicalmed4020093 PMC6630518 31207897

[24] Rwanda Biomedical Centre RBC. Neglected Tropical Diseases Strategic Plan 2019-2024. Kigali: Rwanda Biomedical Centre (RBC). 2019.

[25] Rujeni N, Mazimpaka A, Tumusiime M, Nyandwi E, Rutayisire G, Kayiranga P, et al. Pre-school aged children are exposed to Schistosoma through Lake Kivu in Rwanda. AAS Open Res. 2019;2:7. doi: 10.12688/aasopenres.12930.1 36419723 PMC9648362

[26] Anderson RM, Cano J, Hollingsworth TD, Deribe-Kassaye K, Zouré HGM, Kello AB, et al. Responding to the cuts in UK AID to neglected tropical diseases control programmes in Africa. Trans R Soc Trop Med Hyg. 2023;117(3):237–9. doi: 10.1093/trstmh/trac109 36416069 PMC9977241

[27] ALMA. Rwanda’s fight against neglected tropical diseases: a blueprint for regional success. 2025. https://alma2030.org/news/rwandas-fight-against-neglected-tropical-diseases-a-blueprint-for-regional-success/#:~:text=A%20cornerstone%20of%20Rwanda’s%20strategy,roadmap%20for%20overcoming%20these%20barriers

[28] Rwanda Biomedical Centre. World NTD Day 2021: An Overview of Neglected Tropical Diseases. 2021. https://rbc.gov.rw/marburg/world-ntd-day-2021-an-overview-of-neglected-tropical-diseases/#:~:text=Remarkable%20progresses%20were%20made%20to,NTDs%20as%20public%20health%20problems

[29] Lu C, Chin B, Lewandowski JL, Basinga P, Hirschhorn LR, Hill K, et al. Towards universal health coverage: an evaluation of Rwanda Mutuelles in its first eight years. PLoS One. 2012;7(6):e39282. doi: 10.1371/journal.pone.0039282 22723985 PMC3377670

[30] RSSB. CBHI scheme: Giving you happiness at best rate. 2024. https://www.rssb.rw/scheme/cbhi-scheme2024

[31] Woldemichael A. The impacts of community-based health insurance on poverty reduction. African Development Bank Group. 2020.

[32] Ortu G, Williams O. Neglected tropical diseases: exploring long term practical approaches to achieve sustainable disease elimination and beyond. Infect Dis Poverty. 2017;6(1):147. doi: 10.1186/s40249-017-0361-8 28950893 PMC5615470

[33] McIntyre D, Thiede M, Birch S. Access as a policy-relevant concept in low- and middle-income countries. Health Econ Policy Law. 2009;4(Pt 2):179–93. doi: 10.1017/S1744133109004836 19187569

[34] WHO. Service availability and readiness assessment (SARA). 2024. https://www.who.int/data/data-collection-tools/service-availability-and-readiness-assessment-(sara)

[35] Albonico M, Levecke B, LoVerde PT, Montresor A, Prichard R, Vercruysse J, et al. Monitoring the efficacy of drugs for neglected tropical diseases controlled by preventive chemotherapy. J Glob Antimicrob Resist. 2015;3(4):229–36. 27842865 10.1016/j.jgar.2015.08.004 PMC5642864

[36] The Economist Intelligence Unit. Breaking the cycle of neglect: Reducing the economic and societal burden of parasitic worms in sub-Saharan Africa. 2021. https://www.EIU_EndFund_report-Complete.pdf

[37] United Nations. 2015. https://www.un.org/sustainabledevelopment/health/

[38] Pabalan N, Singian E, Tabangay L, Jarjanazi H, Boivin MJ, Ezeamama AE. Soil-transmitted helminth infection, loss of education and cognitive impairment in school-aged children: A systematic review and meta-analysis. PLoS Negl Trop Dis. 2018;12(1):e0005523. doi: 10.1371/journal.pntd.0005523 29329288 PMC5766095

[39] Musiega A, Tsofa B, Nyawira L, Njuguna RG, Munywoki J, Hanson K, et al. Examining the influence of budget execution processes on the efficiency of county health systems in Kenya. Health Policy Plan. 2023;38(3):351–62. doi: 10.1093/heapol/czac098 36367746 PMC10074769

[40] Kassahun A, Theodros G, Abebe B, Atkure D, Mekonnen T, Habtamu T, et al. Neglected Tropical Diseases (NTD) service availability at health facilities in Ethiopia: Evidence from 2014 Ethiopian service provision assessment. J Health Dev. 2017;31(Special Issue):378–83.

[41] Ooms GI, van Oirschot J, Okemo D, Waldmann B, Erulu E, Mantel-Teeuwisse AK, et al. Availability, affordability and stock-outs of commodities for the treatment of snakebite in Kenya. PLoS Negl Trop Dis. 2021;15(8):e0009702. doi: 10.1371/journal.pntd.0009702 34398889 PMC8389522

[42] Tefera BB, Tafere C, Yehualaw A, Mebratu E, Chanie Y, Ayele S, et al. Availability and stock-out duration of essential medicines in Shegaw Motta general hospital and Motta Health Centre, North West Ethiopia. PLoS One. 2022;17(9):e0274776. doi: 10.1371/journal.pone.0274776 36112721 PMC9481020

[43] Ethiopian Public Health Institute. Ethiopia Service Availability and Readiness Assessment (SARA). Ethiopia Public Health Institute. 2016. http://www.ephi.gov.et

[44] Gerhardt R, Valiati JF, Canto dos Santos JV. An Investigation to Identify Factors that Lead to Delay in Healthcare Reimbursement Process: A Brazilian case. Big Data Research. 2018;13:11–20. doi: 10.1016/j.bdr.2018.02.006

[45] Nasage NN. The Effects of Delays in Reimbursement of Claims by National Health Insurance Authority on Financial Management of Health Care Facilities in Brong Ahafo Region of Ghana. TIJMG. 2020;103–14. doi: 10.21522/tijmg.2015.se.19.02.art012

[46] World Health Organization. Rwanda’s primary health care strategy improves access to essential and life-saving health services. 2022. http://www.who.int

[47] Chanda P, Hamainza B, Moonga HB, Chalwe V, Pagnoni F. Community case management of malaria using ACT and RDT in two districts in Zambia: achieving high adherence to test results using community health workers. Malar J. 2011;10:158. doi: 10.1186/1475-2875-10-158 21651827 PMC3121653

[48] Counihan H, Harvey SA, Sekeseke-Chinyama M, Hamainza B, Banda R, Malambo T, et al. Community health workers use malaria rapid diagnostic tests (RDTs) safely and accurately: results of a longitudinal study in Zambia. Am J Trop Med Hyg. 2012;87(1):57–63. doi: 10.4269/ajtmh.2012.11-0800 22764292 PMC3391058

[49] van Doorslaer E, O’Donnell O, Rannan-Eliya RP, Somanathan A, Adhikari SR, Garg CC, et al. Effect of payments for health care on poverty estimates in 11 countries in Asia: an analysis of household survey data. Lancet. 2006;368(9544):1357–64. doi: 10.1016/S0140-6736(06)69560-3 17046468

[50] Wagstaff A, Eozenou F, Smitz M. Out-of-Pocket Expenditures on Health: A Global Stock Take. Washington, D.C.: The World Bank. 2019.

[51] Dong Z, Tao Q, Sun G. Survey and analysis of the availability and affordability of essential drugs in Hefei based on WHO / HAI standard survey methods. BMC Public Health. 2020;20(1):1405. doi: 10.1186/s12889-020-09477-9 32933517 PMC7493966

[52] Saxena S, Aatik A, Syed A, Noopur G. Factors influencing women’s access to healthcare services in low- and middle-income countries: A systematic review. NURSEARCHER. 2023;3(02).

[53] Berki SE. A look at catastrophic medical expenses and the poor. Health Aff (Millwood). 1986;5(4):138–45. doi: 10.1377/hlthaff.5.4.138 3102333

[54] Girma F, Jira C, Girma B. Health services utilization and associated factors in Jimma zone, South west Ethiopia. Ethiop J Health Sci. 2011;21(Suppl 1):85–94. 22435012 PMC3275873

[55] Goudge J, Gilson L, Russell S, Gumede T, Mills A. Affordability, availability and acceptability barriers to health care for the chronically ill: longitudinal case studies from South Africa. BMC Health Serv Res. 2009;9:75. doi: 10.1186/1472-6963-9-75 19426533 PMC2694171

